# Mr. Davies, on Candour in the Vaccine Contest

**Published:** 1809-02

**Authors:** J. Davies


### *To the Editors of the Medical and Physical Journal.*

GENTLEMEN,

ALTHOUGH not unconscious that any additional observations on the long contested subject of Vaccination, will be deemed by some superfluous, and by most unnecessary; yet there is something, I think, so instinctively important in a subject, which comprehends the security of mankind from the ravages of a destructive and pestilential disease, that in my opinion I can adduce no plea more forcible as an apology for the intrusion of the present unconnected remarks upon your liberal and candid attention, and which, I beg leave, with the utmost diffidence, to recommend, not as dictated by the bigotted voice of blind enthusiasm, but as the genuine and impartial result of an unprejudiced investigation, dispassionate enquiry, and candid observation.

It is to be sincerely regretted, that the interests of vaccination have sustained such considerable injuries from the partial, uncandid, and bigotted point of view, in which numbers have taken up a subject pregnant with such incalculable importance to the community at large, and instead of acquitting themselves of a point of duty, which society had a right to expect, by carefully investigating, and fairly deciding the question, have embraced sentiments wholly inimical to the genuine interests of an invaluable discovery, merely because their minds were unfortunately poisoned by the distasteful bias of unreasonable prejudices.

Nor am I less of opinion, on the other hand, that numbers have been carried away by the popular current of public approbation, and without once candidly examining the intrinsic value of the cause which they so zealously profess to espouse, have calmly submitted to a disgraceful infringement on the exercise of private judgment, actuated by the same unworthy motives, which would allow them, on any occasion, to become the servile dupes of any novel innovations; insomuch, that I fear it still remains a question not altogether unmeriting attention, whether the vaccine interest has been more weakened by the unnecessary interference, ill-timed exertions, and indiscreet zeal of those who profess themselves its most sincere advocates, or the bold attacks, severe opposition, and unground-ed prejudices of avowed enemies? In attempting, however, a decision of this intricate and important question, we should should have an especial regard that our minds are equally uninfluenced by the favourable sentiments of its professed supporters, or the hostile views of its avowed opposers; and when thus prepared to investigate the subject, the want of candid moderation on the one part, will appear to be so equally counterbalanced by the marked imprudence of the other, that to draw an accurate line of distinction will become a task of no inconsiderable difficulty.

Waving, however, any unnecessary comments, by way of eulogium, allow me, gentlemen, to solicit the exercise of your accustomed candour, whilst I attempt to offer a few fugitive observations, with respect to the unquestionable efficacy of cow-pox protection, as a *general* security against variolous infection, and take the liberty to propose for the attentive consideration of the friends of vaccination, a plan for the regulation of that mode of conduct, by the result of which they will undoubtedly afford additional support to the interest of this truly valuable discovery, by tending to silence the formidable voice of opposition, and place the practice of vaccination upon a firm and unshaken basis.

The method by which the Jennerian Society has attempted to divest the public mind of every spark of prejudice, which it might have unfavourably imbibed on the occasion, is altogether inconclusive, and only calculated to kindle that flame of opposition, which it was doubtless intended to check. Is it (for instance) at all consistent with the dictates of common reason to suppose, that men so blinded by vulgar prejudices, so wilfully deaf to the voice of conviction, as those nominated in the "Deputation," should candidly admit the existence of any thing like failure, in a practice which they have made it their peculiar business to hold out as infallible? Or even if they had acted with candour and impartiality on the occasion, is it not equally unwarrantable to expect that the mere "ipse dixit" of two individuals (acknowledged champions for the cause) should have weight sufficient to undeceive the public mind, on a subject where each claims the peculiar prerogative of judging for himself? The "Deputation," therefore, has been extremely partial, and as such highly unsatisfactory. What reasonable objection could the committee have framed against selecting for the occasion two parties of *contrary* opinions? The question would by this method have arrived at some decision, and the vaccine interest have been unquestionably strengthened.

Though

Though I consider Dr. Rowley's cases as the unconnected effusions of a mind poisoned by extravagant opinions; yet the public will think some attention due to the learned author of "*Schola nova medicinæ.*"

And if, in addition to his observations, we add the candid testimony of several respectable practitioners, who have even taken a decided part in favour of the practice, (particularly that related by Mr. Hardy in the 115th Number of your valuable Journal) I think the occasional occurrence of failures, must appear sufficiently obvious to every mind which excessive partiality has not rendered callous to the force of conviction. It is, however, but an act of justice to observe, that these cases occur very rarely, and even in those solitary instances where the influence of previous vaccination has failed to render the constitution unsusceptible of subsequent variolous contagion. I am perfectly satisfied, that it has at least left the constitution in possession of an influence, which renders it secure from that virulence which evidently constitutes the most alarming feature of the disease. There is also much reason to suspect, that the number of *supposed* failures has been considerably augmented by the injudicious conclusions of several practitioners, who have unwarrantably pronounced their patients secure, before a regular and complete vaccination had taken place; and in this remark I am the more confirmed, from a full conviction, that in the earlier stages of the discovery, several thousands were inoculated for cow-pock by persons totally unacquainted with the nature of the disease, who consequently could not be competent judges of the result; but since its character has become more clearly understood, the number of its opposers have been gradually diminishing, and the success of the practice daily gaining ground.

It has been alleged, with equal impropriety and injustice, by the enemies of vaccination, that the practice is productive of various eruptions, painful to the patient and injurious to the constitution. To this imputation, as unfounded, I enter my caviat. That numerous eruptions (totally unknown before) have made their appearance in the constitution, subsequent to the vaccine infection, is readily allowed by every candid observer, but this may be easily accounted for without any imputation against vaccination.

Another stale and hackneyed objection urged by the Anti-Jennerians, is, that it is impossible to foresee the "Danger of introducing a bestial humour into the human frame," an objection almost too ridiculous to merit any re-

 ply.

ply. If the influence of a "Bestial Humour," indisputably milder in its nature, more benign in its progress, and safer in its consequences, can be incontrovertibly proved to secure the constitution in a general way from the attack of a loathsome, virulent, and dangerous disease, what but the most culpable ignorance could act as an inducement to adopt such a childish, such an unwarrantable argument?

Lest, however, I should encroach upon the limits of your plan, I shall endeavour to confine my few remaining observations, merely to that line of conduct on the part of the vaccine advocates, which I humbly conceive to be most conducive to the interest of this invaluable practice. With this view, therefore, I would cordially recommend them, in the first place, to moderate their pretensions. On subjects of general interest, it is a moral duty incumbent on every individual, who interests himself in their investigation, to pay an implicit attention to the decisive testimony of unprejudiced experience, as well as publicly to avow the result of such experience with unbiassed candour.

By giving up the idea of *universal* protection, and placing the vaccine practice, with respect to occasional failures, upon the footing of variolous contagion, (which has been clearly proved to occur twice in the same subject) the question would have been fairly stated, and no inducement could possibly have existed, to conceal either by open denial or indirect evasion, those existing circumstances, which from the present mode of proceeding, have given rise to such disgusting volumes of useless, petulant, and uninstructive controversy.

Another maxim which I would strenuously endeavour to recommend to their attention, is to avoid scurrilous retaliation; and I think the disgusting method in which most of the controversial papers on this important subject have lately been conducted, will furnish a sufficient plea for urging the present remark. On medical topics of acknowledged import, every writer has not only an undoubted privilege of exercising individual judgment, opposing ungrounded prejudices, and counteracting erroneous notions, but is likewise fully justified in attempting to establish those axioms, which he conceives to be built upon the basis of actual experience; neither ought he on this account, to be at all exposed to the unsparing censure of those with whom he may not happen to coalesce. But how frequently vulgar declamation has been substituted for convincing argument, and a stile of the lowest scurrility resorted to, whereby whereby the author's characters, as gentlemen, and the honour of medicine as a science, have been considerably degraded, you, Sirs, cannot possibly be unaware! But as this disgraceful conduct is by no means confined to the *friends* of vaccination, they are still in a capacity to adopt an infallible antidote against the malicious spleen, illiberal aspersions, and vulgar ridicule of its professed opposers, by treating all the puerile attempts of their feeble malice, not with a disgraceful return of scurrilous language, but with that more powerful, more honourable, and more efficient weapon, silent contempt.

With a few observations, intended to stimulate the vaccine advocate to distinguish between prudent zeal and blind enthusiasm, I mean to conclude the present remarks. Some zealous enthusiasts in this novel practice, have materially (though inadvertently) injured the cause, by vainly attempting to refute every frivolous objection, which the active efforts of designing ignorance might suggest; and whilst some have appeared conspicuously employed in supporting the vaccine interest, before the refined ordeal of a Public Debating Society; others, with an equal share of ignorant enthusiasm, whose professional avocations, in a more important sphere, were sufficient to have engaged their attention, have been widely overstepping all prescribed bounds of propriety and decorum, by unwarrantably intruding upon the duties of *another* profession, and, under a cloak of philanthropy, have equally diminished their own reputation, and encroached upon the business of the regular practitioner. These, and similar effects of the misguided zeal of vaccine enthusiasts, have, I am convinced, been of more serious detriment to the success of the practice, than seems to be generally imagined; but as reform is yet practicable, I flatter myself with a hope, that these hints will not be altogether disregarded.

I am, &c.

J. DAVIES.

